# More than an Incidentaloma: The Nonreportable NIPT

**DOI:** 10.1155/2022/2496057

**Published:** 2022-04-30

**Authors:** Allison M. Jay, Brian Mason, Daniel Lebovic, Paul Chuba

**Affiliations:** ^1^Ascension St. John Genetics, 19229 Mack Ave, Grosse Pointe, MI 48236, USA; ^2^Ascension St. John Obstetrics and Gynecology, 22151 Moross Rd I, Suite, Grosse Pointe, MI 48236, USA; ^3^Ascension St. John, Hematology and Oncology, 19229 Mack Ave, Grosse Pointe, MI 48236, USA; ^4^Ascension St. John Macomb, Radiation Oncology, 11800 Twelve Mile Rd, Warren, MI 48093, USA

## Abstract

Noninvasive prenatal testing (NIPT), which utilizes a maternal blood sample to detect fetal gender and screen for fetal aneuploidy (abnormal chromosomes), is widely used in obstetrics to screen for Trisomies 21, 18, and 13. Per the literature, approximately 0.3% of pregnant woman's results are nonreportable. Reasons include low fetal fraction, insufficient DNA, vanishing twin, twin pregnancy, clonal mosaicism, and maternal neoplasia. Here, we describe a 25-year-old G2P1 pregnant woman who had two nonreportable NIPT results and subsequently was diagnosed with lymphoma. We discuss the importance of clinical exam in correlation with the results to offer comprehensive evaluation of the patient with a nonreportable finding, given malignancy occurs in 1/1000 pregnant women. This report overviews proposed management guidelines for pregnant women with a nonreportable result and helps to address discomfort the treating physician may feel in discussing this result with their patient.

## 1. Background

Noninvasive prenatal testing became readily available in the United States and Western Europe in 2011. As opposed to amniocentesis which is considered an invasive diagnostic test, this screening testing involves taking a tube of the pregnant patient's blood as early as 10 weeks. While NIPT is used worldwide, the literature suggests that many pregnant woman may misunderstand the difference between the words “diagnostic” and “screening.” Quaresima et al. evaluated 325 pregnant women attending an antenatal clinic and found that 34.3% of women who chose cell-free fetal DNA testing incorrectly considered it diagnostic and furthermore highlight that this misunderstanding can be dangerous for care [1].

NIPT uses genome sequencing to analyze cell-free fetal DNA to assess if the pregnancy is at risk for chromosomal disorders such as Down's syndrome, Trisomy 13, or Trisomy 18. While this test was initially only offered to women with advanced maternal age older than 35, the American College of Obstetricians and Gynecologists issued a new guideline in 2020 recommending that this screening should be offered to all pregnant individuals. In rare instances, this testing may yield multiple chromosomal abnormalities which are due to abnormalities shed by cancer cells. The present case illustrates the importance of clinicians being aware of this implication for early cancer detection and treatment.

## 2. Case

A 25-year-old Caucasian G2P1 female was evaluated by the Maternal Fetal Medicine (MFM) Consultant as she was considered a high-risk pregnancy due to a history of premature labor in her prior pregnancy. In addition, she had cervical incompetence and had cervical cerclage. The patient had no history of fibroids, or autoimmune, or other illnesses. Past medical history was remarkable for an appendectomy.

As part of screening, a LabCorp Materni21 NIPT was sent, and the results came back nonreportable. Eleven days later, a second sample was sent, and the result came back nonreportable. The lab noted that the results were globally aberrant with the following abnormalities: partial Trisomy 1, partial monosomy 3, 1q duplication, 3 p elevation, large interstitial deletion of 4q, elevation of chromosome 5, deletion of 7q, increase chromosome 9, monosomy 11, Trisomy 14, monosomy 15, Trisomy 16, 19 large duplication, trisomy of chromosome 20, and monosomy 22. Amniocentesis was performed and showed normal 46xx karyotype, and chromosomal microarray was normal.

**Figure 1:**
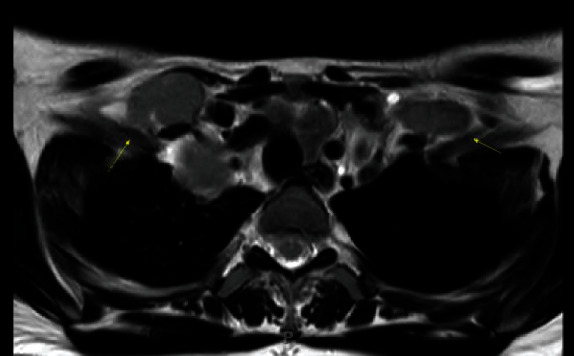
T2-weighted sequence: bilateral supraclavicular lymphadenopathy.

**Figure 2:**
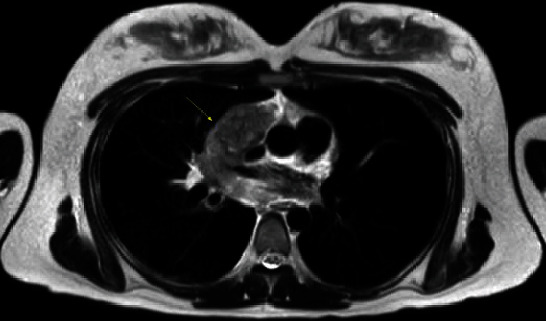
T2-weighted sequence: anterior mediastinal mass consisting of matted lymphadenopathy.

One month after those results, the patient presented at 20 weeks to her MFM consultant and reported noticing her right and left supraclavicular lymph nodes had increased in size and were tender to palpation. On review of systems to evaluate for any signs of illness, she reported dysphagia and dyspnea. At our community hospital, the geneticist was contacted. She then reached out to the chief oncologist and made him aware of the literature suggesting that multiple aneuploidies could be associated with malignancy, after which the patient was promptly evaluated. The patient soon thereafter had a MRI of the chest/neck and abdomen/pelvis and an US-guided biopsy of the right supraclavicular lymph node (Figures 1 and 2, MRI of chest/abdomen). The MRI of the chest/neck demonstrated extensive bulky and matted mediastinal lymphadenopathy. Involvement of several nodal stations was identified including hilar, pretracheal, subcarinal, prevascular, paratracheal, and supraclavicular adenopathy.

Pathology results were consistent with classical Hodgkin's lymphoma. The patient chose to continue the pregnancy and was started on a chemotherapy regiment of doxorubicin, bleomycin, vinblastine, and dacarbazine. The patient delivered a healthy baby and has subsequently done well with close follow-up in hematology/oncology.

## 3. Discussion

### 3.1. History of the Nonreportable Result

Using cell-free fetal DNA in the circulating plasma of pregnant women to screen for fetal aneuploidies was first available in 2011. Starting in 2013, reports in the literature emerged of “false positives” of women who had positive NIPT testing, but the fetal karyotype or chromosomal microarray was normal. Early on, the working diagnosis for these was either confined placental mosaicism or a twin demise [2, 3].

Gradually, more reports in the literature occurred citing malignancy as another cause of such results. The most common cancers in pregnancy include Hodgkin's lymphoma, non-Hodgkin's lymphoma, breast cancer, cervical cancer, ovarian cancer, colorectal cancer, and leukemia.

With some of these cancers, the tumor DNA is circulating in the maternal circulation. Because the tumor DNA has multiple regions of duplication and deletion, this causes multiple abnormalities in the NIPT (Plon, Biancchi). Monosomy results are sometimes found which would not be anticipated to occur in a fetus.

In 2015, Bianchi et al. [2] published a retrospective analysis of detailed clinical and genome-wide sequencing data from eight women who had NIPT results positive for an aneuploidy. They found that having more than one aneuploidy results was more often associated with cancer. Of the eight patients they evaluated who had abnormal NIPT, seven had normal karyotypes upon workup. The cancers they observed included neuroendocrine, non-Hodgkin's B cell lymphoma, colorectal, Hodgkin's lymphoma, and acute T cell lymphoblastic leukemia. As time progressed, more studies have been published evaluating the positive predictive value of having a NIPT result with multiple aneuploidies and how to improve diagnostic accuracy.

In 2016, Yaron [4] published “The implications of non-invasive prenatal testing failures” a review of underdiscussed phenomenon. Yaron noted that because of the high reported sensitivities of >99.9%, many patients and care providers felt that NIPT testing was an “infallible test.” He emphasizes an important point that while sensitivities and specificities are indeed important, even more essential are the positive predictive value, which reflects a positive result being a true positive, and the negative predictive value, which is the chance of a negative test result being a true negative.

While these markers are often touted by companies, he notes that an often-overlooked metric is the no-call result or test-failure rate. Usually, the causes of a no-call result include the following primary reasons: (A) sample collection labeling, (B) low fetal fraction < 4%, and (C) assay failure including problems with DNA extractions, amplification, and sequencing (Yaron). For these, ACOG had recommended considering a repeat blood sample. However, redraws may be less than 30% efficient (Yaron), and especially in the case of maternal aneuploidies, it may be more prudent to follow workup as outlined below.

**Figure 3:**
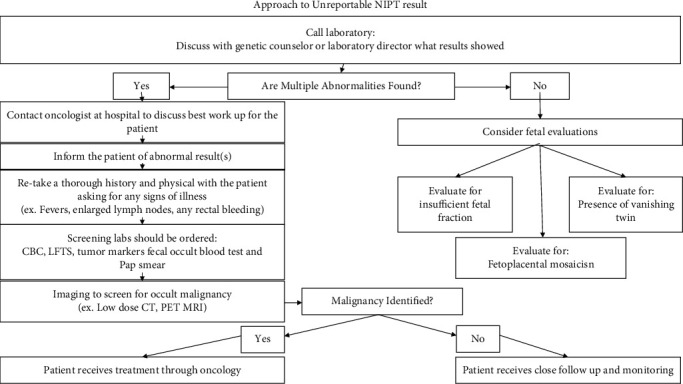


In 2019, Ji et al. [5] described their multicenter retrospective analysis evaluating 639 pregnant women who tested positive for multiple chromosomal aneuploidies on noninvasive prenatal screening (NIPS) testing. They used a novel bioinformatics algorithm called the cancer detection pipeline (CDP). Their results showed that multiple chromosomal aneuploidies had a positive predictive value of 7.6% for maternal malignancy. When they combined their CDP with plasma tumor makers, this gave a positive predictive value of 75% [5]. While the CDP model may not be widely available yet, this finding highlights the importance of considering tumor markers to further evaluate a NIPT result with multiple aneuploidies (Figure 3).

Lenaerts et al. [6] at the University Hospital Leuven also published work on how they improved the positive predictive value using an NIPT analysis pipeline they called GIPseq. They presented a multidisciplinary care model for efficient treatment of patients. They note before their study that there were no published posttest evaluation of pregnant woman who were thought to have cancer because of their NIPT result. While smaller community hospitals may not have the pipelines of Jie et al. and Lenaerts, these hospitals may be able to establish protocols for clinical follow-up when NIPT is concerning for maternal malignancy. It may be prudent for the maternal fetal medicine physician or obstetric and gynecologist department to reach out and have a multidisciplinary on call team that includes genetics and oncology that could be activated in the event of an abnormal NIPT with multiple chromosomal aneuploidies.

### 3.2. Handling the NIPT-Standardized Approach

In 2018, Dr. Carlson [7] published a table entitled “Stepwise Evaluation of the Patient with More than One Cell-Free DNA” including taking history and physical, ordering CBC to evaluate for leukemias, LFTS to evaluate for liver cancer, fecal occult blood to evaluate for colon, and chest X-ray to evaluate for lung and mediastinal masses. In June 2021, Dow et al. [8] published an updated proposed investigation guidelines following abnormal NIPT results concerning for malignancy. They recommend multidisciplinary input as outlined, then maternal investigations including history and clinical exam asking about fatigue, weight loss, pain, enlarged lymph nodes, and tests including tumor markers, CBC, liver function tests, and chromosomal microarray, and targeted evaluation of suspected site of malignancy if identified. If there is no site identified, they recommend general imaging CXR and breast and abdominal US and consider low-dose PET-CT and PET-MRI. If no maternal malignancy is identified, there should be close monitoring and repeat cell-free fetal DNA testing postpartum [8].

### 3.3. Ethical/Practical Implications

Academic centers may have access to pipelines which may improve the positive predictive value and help better guide how they frame an abnormal nonreportable result. For physicians at smaller hospital who have limited technology, the guidelines by Carleson and Dow may offer a standardized approach to evaluating the nonreportable NIPT result. Having a protocol in place for obstetrics/gynecology physicians may help with discomfort felt in addressing a potential cancer diagnosis in their patient given the NIPT test is not designed as a cancer screening test. In a survey of over 300 genetic counselors, half of genetic counselors would be uncomfortable providing nonreportable NIPT results and the potential association with malignancy, and 91% felt that having national guidelines in place would help standardize reporting of these findings [9]. For the provider who gets a nonreportable result, it is important to have a framework in which to evaluate the patient. Dow et al. outline a framework in their paper. It is important to note while with other laboratory results, where repeating may lead to normalization of the value, with nonreportable results, this is often not the case and repeating the draw may lengthen time to diagnosis for the mother with cancer [10].

In conclusion, for the patient who has a nonreportable result, it is prudent to review the clinical history and consider a comprehensive cancer evaluation. It is important for treating obstetrics and gynecologists to know how to work up the nonreportable NIPT result and approach discussing this result with their patient. For physicians offering this testing, the availability of checklists with Dow's proposed management guidelines or Carleson's Stepwise approach to attach to the patient chart may help standardize the patient discussion about the implications of this “incidental” finding.

## Data Availability

No underlying data were used to support this study.
